# Spinal cord compression as tumor onset: an unusual case report of Hodgkin lymphoma in a teenager

**DOI:** 10.1186/s12887-021-02834-6

**Published:** 2021-08-24

**Authors:** Giulia Salomone, Milena La Spina, Giuseppe Belfiore, Gregoria Bertuna, Laura Cannavò, Stefano Catanzaro, Salvatore D’Amico, Mariaclaudia Meli, Andrea Musumeci, Lucia Salvatorelli, Maria Grazia Scuderi, Corrado Spatola, Mariella Valenzise, Andrea Di Cataldo, Giovanna Russo

**Affiliations:** 1 Postgraduate Training Program in Pediatrics, Department of Clinical and Experimental Medicine, University of Catania, Catania, Italy; 2 Unit of Pediatric Hematology Oncology, Department of Clinical and Experimental Medicine, Hospital Policlinico “G. Rodolico”, University of Catania, Via Santa Sofia 78, Catania, Italy; 3 Unit of Radiology, Department Ingrassia, Hospital Policlinico “G. Rodolico”, University of Catania, Catania, Italy; 4 Unit of Pediatrics, Hospital Policlinico “G. Martino”, University of Messina, Messina, Italy; 5 Unit of Anatomic Pathology, Department Ingrassia, Hospital Policlinico “G. Rodolico”, University of Catania, Catania, Italy; 6 Unit of Pediatric Surgery, Department Ingrassia, Hospital Policlinico “G. Rodolico”, University of Catania, Catania, Italy; 7 Unit of Radiotherapy, Department Ingrassia, Hospital Policlinico “G. Rodolico”, University of Catania, Catania, Italy

**Keywords:** Adolescent, Case report, Epidural mass, Hodgkin lymphoma, Spinal cord compression

## Abstract

**Background:**

Spinal cord compression (SCC) is an uncommon, severe complication of Hodgkin lymphoma (HL), occurring in 0.2% of cases at the onset and in 6% during disease progression. We present a teenager with SCC with clinical onset of HL; her pre-existing neurological abnormalities covered the presence of an epidural mass, which could have misled us.

**Case presentation:**

A 13-year-old girl presented with a three-month history of lower back pain and degrading ability to walk. She suffered from a chronic gait disorder due to her preterm birth. A magnetic resonance imaging of the spine revealed an epidural mass causing collapse of twelfth thoracic vertebra and thus compression and displacement of the spinal cord. Histological examination with immunohistochemical analysis of the epidural mass demonstrated a classic-type Hodgkin lymphoma. Early pathology-specific treatment allowed to avoid urgent surgery, achieve survival and restore of neurological function.

**Conclusions:**

Children and adolescents with back pain and neurological abnormalities should be prioritized to avoid diagnostic delay resulting in potential loss of neurological function. SCC requires a prompt radiological assessment and an expert multidisciplinary management.

## Background

Spinal cord compression (SCC) among pediatric patients can be attributed to various underlying conditions, including malignancy. SCC is a rare oncological emergency due to paravertebral benign or malignant tumors, arising from neurogenic, mesodermal, germ cell or lymphatic tissues [1, 2]. Neurologic manifestations related to SCC has been described as uncommon for clinical onset of Hodgkin lymphoma (HL) in adults and less frequently in children [3, 4]. We describe an adolescent with intraspinal epidural HL who presented vertebral collapse and SCC.

## Case presentation

**Fig. 1:**
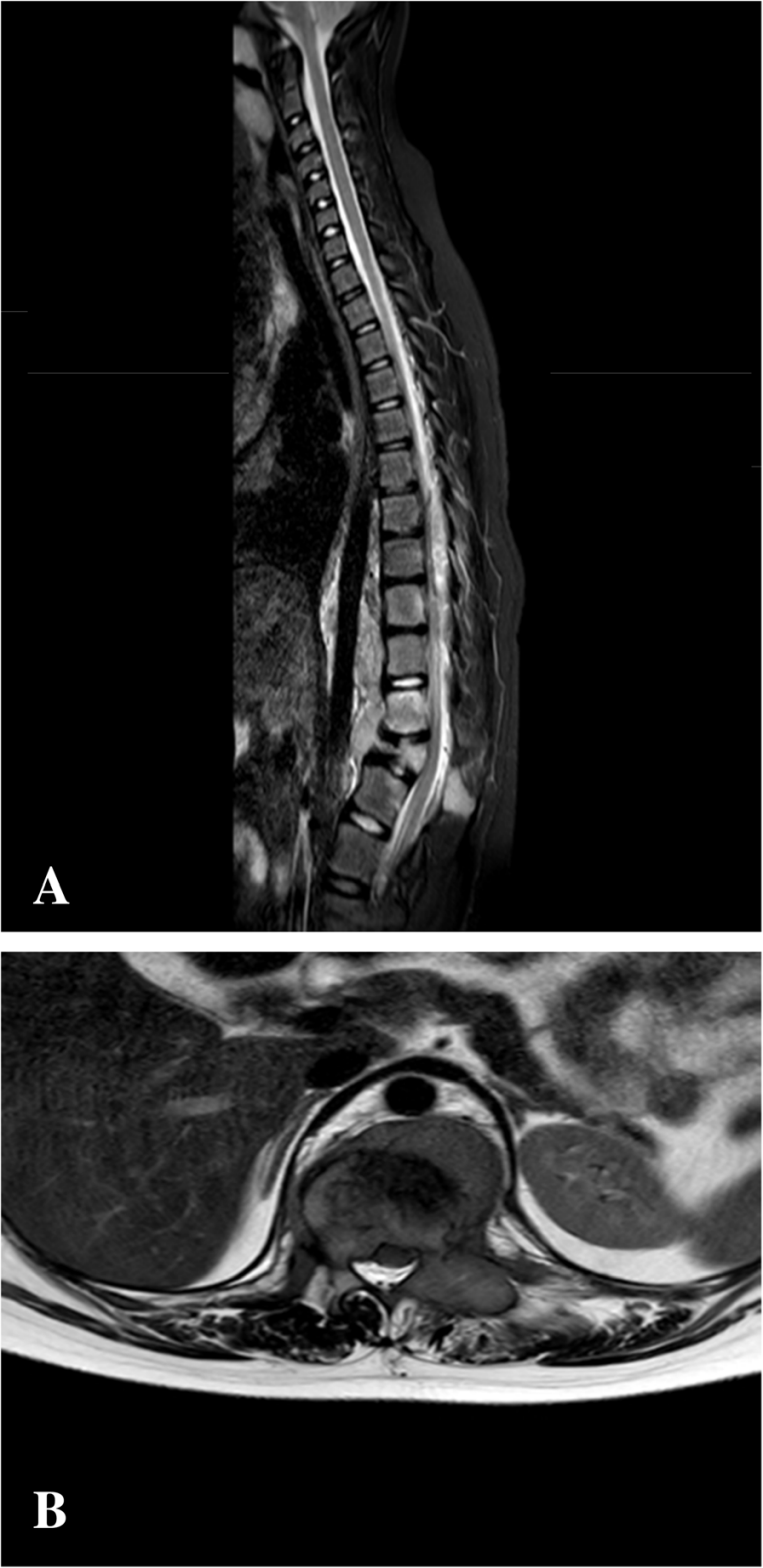
Initial magnetic resonance imaging of the spine. Magnetic resonance imaging of the spine, fat-saturated T2-weighted sagittal plan (**A**) and T2-weighted axial plan (**B**), revealing an epidural mass causing collapse of twelfth thoracic vertebra and compression and displacement of the spinal cord from Th12 to L1

**Fig. 2:**
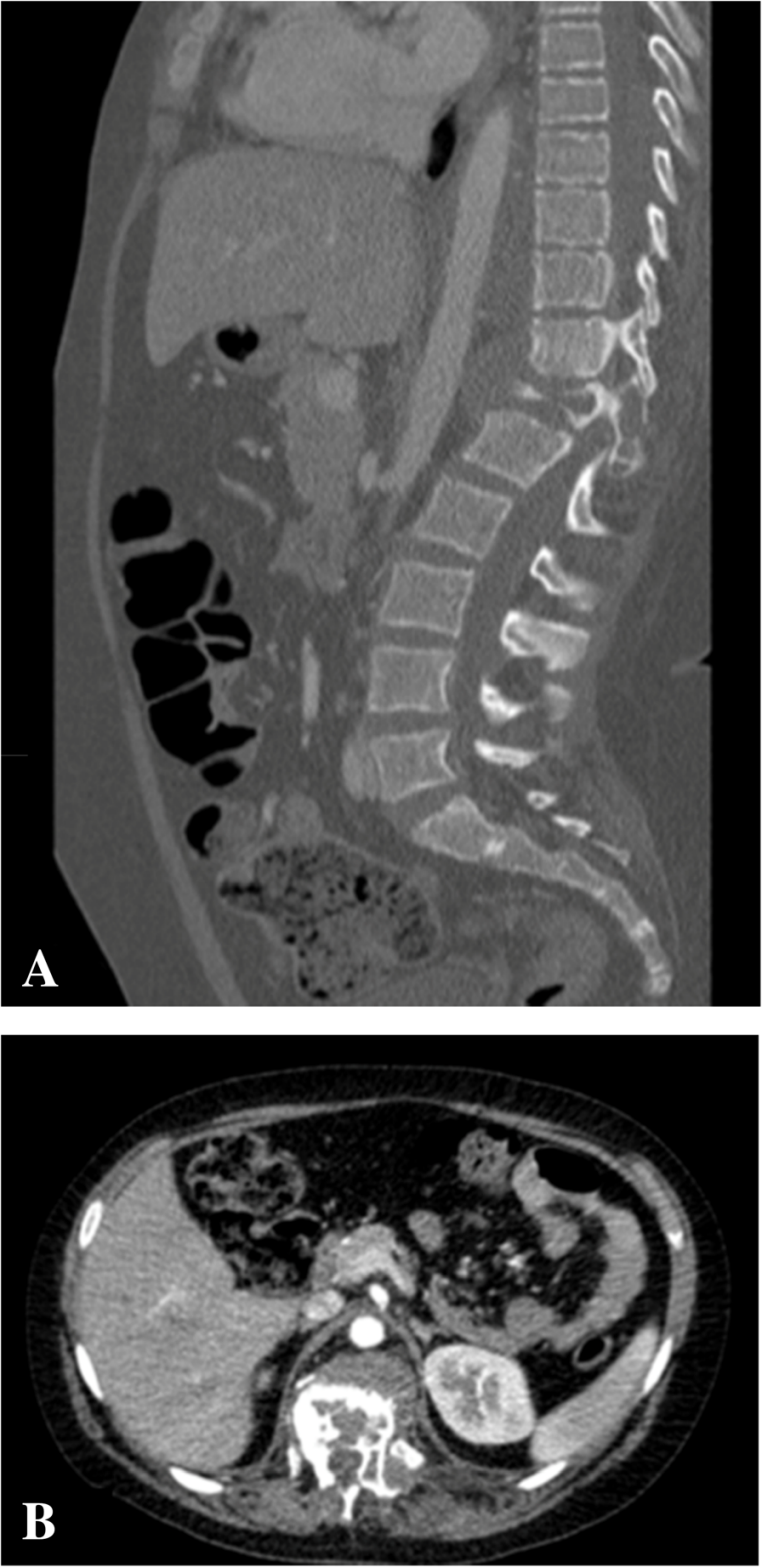
Initial computed tomography scan of chest and abdomen. Computed tomography scan of chest and abdomen, contrast-enhanced multiplanar reformatted (MPR) images in the sagittal (**A**) and axial (**B**) plans, showing a paravertebral mass extended up to Th8 level and to L3 level leading to vertebral collapse at Th12, thinning of the spinal canal and cord compression, and expanding toward retroperitoneal structures

**Fig. 3:**
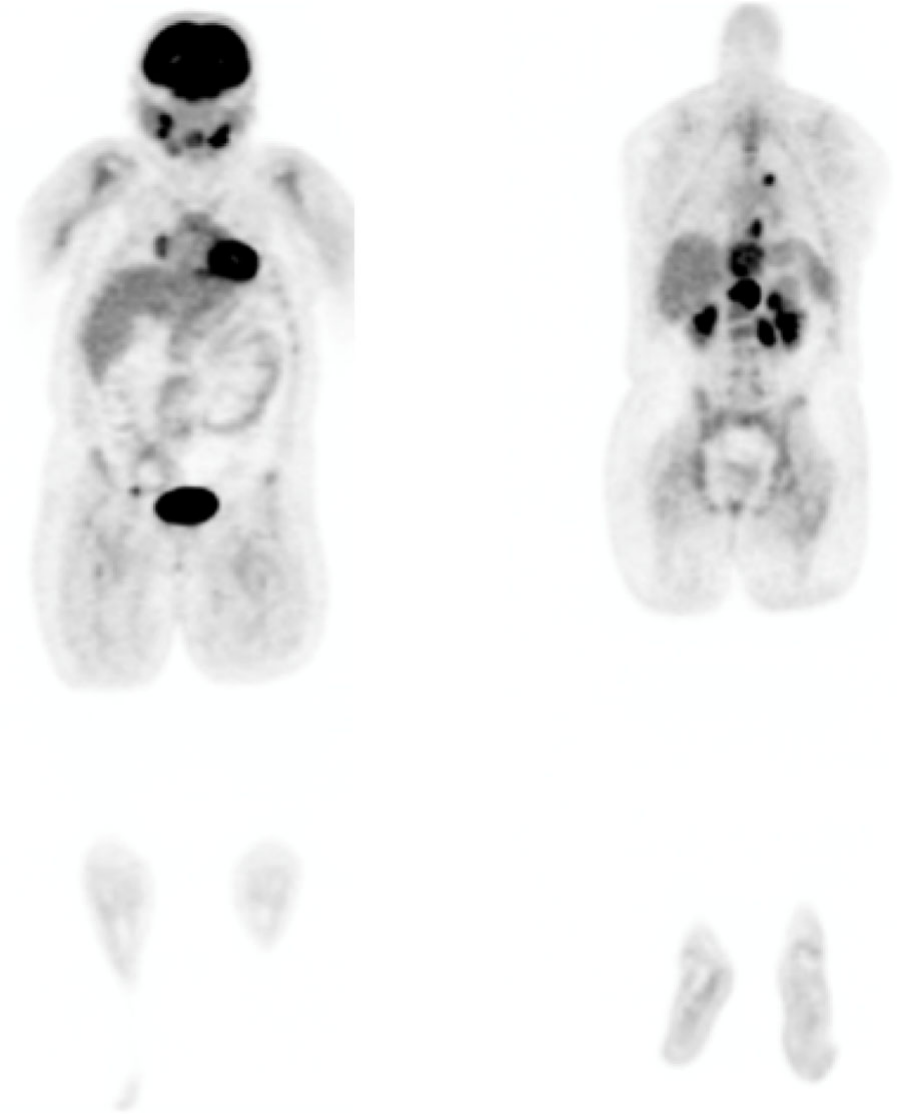
^18^F-fluordeoxyglucose positron emission tomography scan. ^18^F-fluordeoxyglucose positron emission tomography scan showing multifocal FDG uptake involving the epidural mass and lymph nodes in the mediastinum and cervical groups

A 13-year-old girl complained of a three-month history of lower back pain and degrading ability to walk. Her past medical history revealed developmental delay with gait disorder related to premature birth at 31 weeks of gestation. On physical examination, she had mildly enlarged lymph nodes in the cervical group, motor weakness, muscle hypotrophy, loss of sensation and brisk deep tendon reflexes at the lower extremities. Laboratory investigations detected unremarkable peripheral blood cell count, high levels of erythrocyte sedimentation rate (74 mm; normal up to 20 mm) and C-reactive protein (89 mg/L; normal up to 10 mg/L). A peripheral blood lymphocyte typing was negative for presence of blasts and a bone marrow aspiration allowed to exclude tumor involvement. A contrast-enhanced magnetic resonance imaging (MRI) of the brain and the spine showed an epidural mass infiltrating the spinal canal via the left neural foramen. The mass, extending from Th12 to L1 with compression and displacement of the spinal cord (Fig. 1A, B), was causing destruction and collapse at the twelfth thoracic vertebra. To ascertain the disease spread and the correct risk stratification, a total body computed tomography (CT) was performed. The CT confirmed the presence and the extension of the paravertebral mass and revealed multiple enlarged lymph nodes in the mediastinum and cervical groups (Fig. 2A, B). Staging ^18^F-fluordeoxyglucose positron emission tomography (FDG-PET) scan detected pathological FDG uptake involving the paravertebral tumor and the lymph nodes detected at CT scan (Fig. 3). The patient underwent ultrasound-guided biopsy of the epidural mass. Histological examination with immunohistochemical analysis demonstrated a classic-type, nodular sclerosing variant HL, excluding both a primary mediastinal large B cell lymphoma and an anaplastic large cell lymphoma. Considering both bone involvement, due to contiguity of the mass, and neurological symptoms, related to compression by tumor, the girl was classified as stage 4. After informed consent was acquired, she received 2 cycles of chemotherapy using Prednisone, Vincristine, Doxorubicin, Etoposide (OEPA regimen), followed by 4 cycles of Prednisone, Dacarbazine, Vincristine, Cyclophosphamide, Etoposide, Doxorubicin (DECOPDAC-21 regimen), according to the specific international protocol European Network-Paediatric Hodgkin Lymphoma (EuroNet-PHL-C2). During treatment there was a rapid and marked improvement of motor and sensory deficits. Total-body CT after treatment revealed a complete remission of the epidural mass, reduction in lymph node size in the mediastinum group, but no change of the cervical lymph nodes. FDG-PET scan showed an uptake only in the upper neck lymph nodes. Therefore, the patient received a total dose of 2880 cGy radiation therapy delivered to the laterocervical lymph nodes at 180 cGy per fraction over 16 days. CT-scan after radiation therapy documented complete remission.

**Fig. 4:**
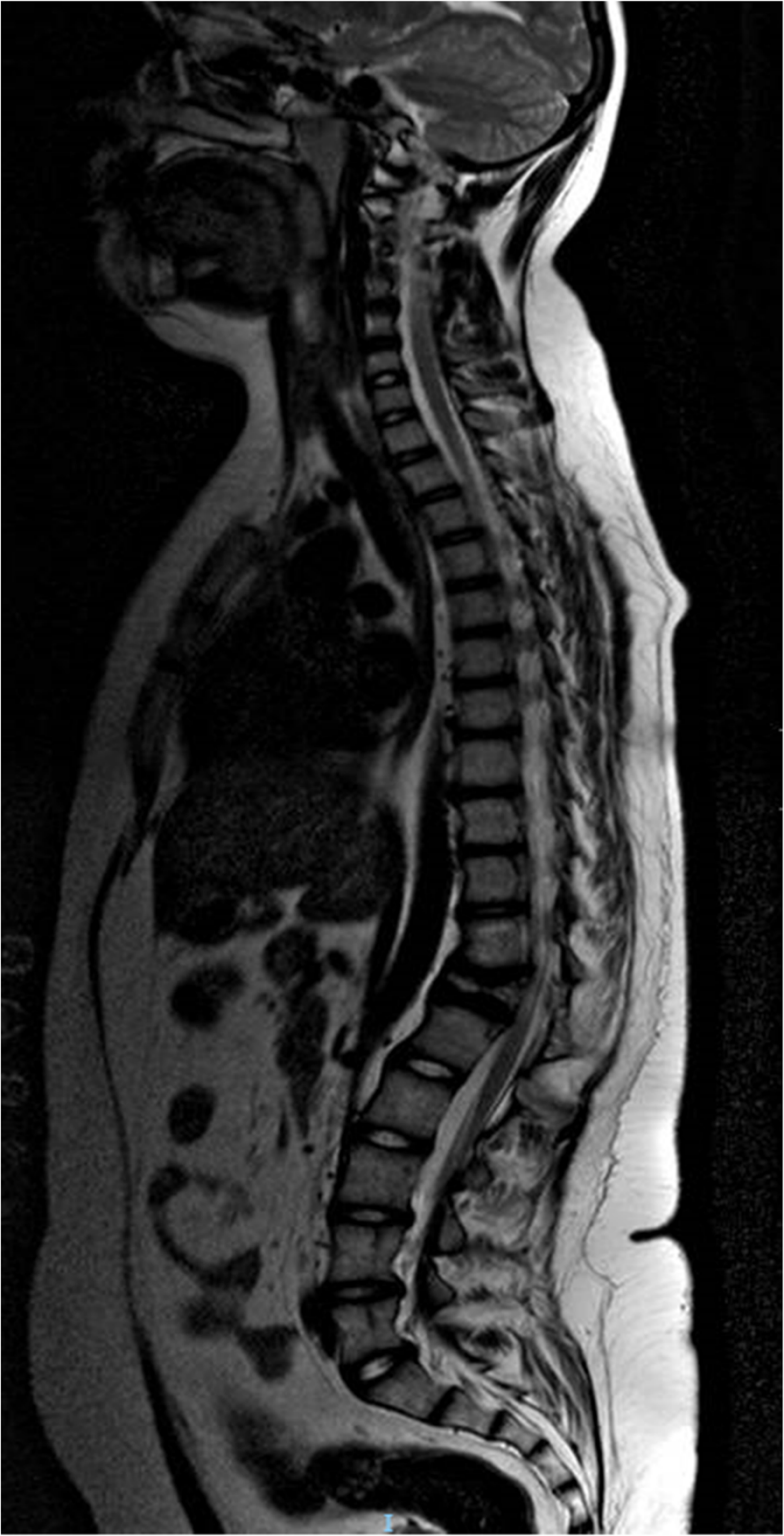
Follow-up magnetic resonance imaging of the spine. Magnetic resonance imaging of the spine, T2-weighted sagittal plan, a year after completion of treatment: residual vertebral collapse at Th12 with finding of “vertebra plana”; no residual tumor mass

The girl was required to wear an orthopedic corset for six months, following the vertebral collapse, to maintain an erect position. Intensive neuromotor rehabilitation improved strength and muscle trophism. She started walking again with assistance initially, until complete ambulatory recovery. She performed clinical and laboratory evaluation, chest x-ray and abdomen ultrasound every three months for 1 year and every four months thereafter. Three years post-treatment, she has no residual back pain or neurological sequelae. MRI acquired annually from tumor onset confirms complete remission, no relapse or complications (Fig. 4).

## Discussion and conclusions

SCC among children and adolescents mostly arises from benign process, but it can also result from tumor. Benign processes comprise trauma, hematoma or infectious disease, as abscess or spinal tuberculosis. Benign tumors include plexiform neurofibroma, hemangioma, angiolipoma and osteoblastoma. Pediatric paravertebral malignant tumors are predominantly neuroblastoma and soft tissue sarcomas, followed by a variety of histologic subtypes including lymphomas, more frequently non-Hodgkin lymphoma (NHL) [1, 5].

SCC increases paravertebral tumor morbidity, that ranges from chronic back pain to sensory, motor and autonomic neurological disorders and paralysis [2, 6]. Functional damage is reported to be significantly associated to tumor type, development time of neurological abnormalities, advancement of disease and treatment modalities [1]. In children, lymphomas have a better outcome than other paravertebral malignant tumors [5].

HL is typically nodal involving lymph nodes in 90% of cases; in 10% of cases, HL may present as an extranodal disease. SCC is a severe complication of HL reported in 6% of cases during disease progression and in only 0.2% at clinical onset [4, 7, 8].

Cases of HL presenting with SCC have been described both in adults and children. We carried out a systematic review of the literature of all pediatric reports. We found 45 articles using the following keywords: “Hodgkin lymphoma”, “spinal cord compression”, “children” and/or “pediatric”. For each article the following restrictions were applied: publications in the last thirty years (20 articles were excluded), patient age 16 years and younger (1 article was excluded) and from publications originating in English (2 articles were excluded). Another 15 articles referred to NHL and were excluded. The remaining 7 articles consisted of: 1 systematic review, 1 review, 3 case series and 2 case reports. Those articles were analyzed to compare pediatric and adolescent patient experiences, symptomatology and histology (Table 1). A total of 16 patients, among children and adolescents (age reported from 8 to 14 years), with SCC due to HL have been described to date. Although clinical and histological details are limited in the evaluated studies, patients with HL and SCC were predominantly males (4 subjects reported) presenting with neurologic complaints [10, 11]. Thoracic and lumbar spine were most commonly involved, while cervical SCC has been found in only one case in a teenage girl [6]. Systemic symptoms occurred only in the 2 subjects described by Gupta et al. [4] The most frequently observed histology subtype was mixed cellularity (3 reported cases), followed by classic subtype, even if the histology subtype of 11 patients is unknown [4, 9].

**Table 1:** Reported case reports and series of SCC due to HL in children and adolescents. All 16 cases of SCC due to HL involving children and adolescent reported to date were analyzed to compare patient experiences, symptomatology and histology

*References*	*Year*	*No. Cases*	*Age (yrs)/Gender*	*Symptoms and Signs*	*Histology subtype*
Klein et al [2]	1991	8	Unavailable	Pain, inability to walk, sphincter dysfunction	Unavailable
Aysun et al [9]	1994	1	10/ Male	Unsteadiness	Mixed cellularity
Pollono et al [10]	2003	2	12/ Unavailable 14/ Unavailable	Pain, paresis/paralysis Pain, paresis/paralysis	Unavailable Unavailable
Gunes et al [1]	2009	1	7/ Unavailable	Pain, motor deficits, autonomic dysfunction	Unavailable
Gupta et al [4]	2009	2	10/ Male 11/ Male	Fever, lymphadenopathy, pain Fever, paraplegia, autonomic dysfunction	Mixed cellularity Mixed cellularity
Baroni et al [11]	2014	1	8/ Male	Pain, paraplegia, sensory loss, sphincter dysfunction	Classic nodular sclerosing
Toto et al [6]	2016	1	12/ Female	Pain, hyposthenia in upper extremities	Classic

Symptoms consistent with an epidural mass depend on the level of the lesion and they may progress slowly. Prolonged back pain or discovery of radicular or nerve damage should be immediately investigated for malignant cord compression with tumor extension into the nerve roots [3, 9]. As in our reported case, pre-existing neurological abnormalities may initially cover these complaints and lead to diagnostic exclusion of malignancy, particularly in the absence of significant lymphadenopathy and general health decay [12]. Nonspecific symptomatology led to initial misdiagnoses of pyelonephritis, Guillain-Barrè syndrome and, in another case, Pott’s disease [4, 13].

MRI of the entire spine is the diagnostic radiological evaluation of choice. FDG-PET scan is essential for staging, risk stratification and identifying patients who inadequately respond to chemotherapy and should receive radiotherapy [14, 15].

HL is a slow-growing, malignant lymphoproliferative disorder and quickly responds to initial medical therapy in most cases [1]. Corticosteroids and chemotherapy should be the first choice of treatment, even in case of SCC, but some sources report early local radiotherapy and surgical debulking [6, 13]. Epidural HL responds favorably to specific chemotherapy and radiotherapy: complete clinical response and functional recovery have been reported in 61% and 86% of cases, respectively [4, 16]. Given the above response, the possibility of spinal instability and growth anomalies, emergency decompressive laminectomy (EDL) is an unnecessary initial treatment of HL causing SCC. EDL should be reserved for advanced disease or relapsed cases with severe, progressive neurological symptoms [1, 17]. Considering the therapeutic EuroNet-PHL-C2 protocol for HL used at the time and the complete clinical response at that site, our patient only received radiotherapy in the upper neck lymph nodes and not in the paravertebral region.

Malignant SCC requires a multidisciplinary approach with experts in pediatric oncology, including a pediatrician, a radiologist, a pathologist, a radiotherapist, a surgeon, a neurosurgeon and a physiotherapist. Our expert multidisciplinary team provided initial targeted care and prevented long-term complications related to radiotherapy and surgical interventions. We carried out a minimally invasive diagnostic technique and began an early pathology-specific treatment avoiding an EDL. We achieved both survival and restoration of neurological functions.

Tumors among adolescents are often missed because they are rare and adolescents, for various reasons, often do not effectively communicate their discomfort from the disease. If a tumor is suspected, adolescents should be directed to a pediatric oncology unit, where management is more age appropriate [18, 19].

SCC is rare in patients with HL; however, it represents an oncological emergency due to consistent rates of mortality and neurological sequelae. Any physician observing children and adolescents with back or radicular pain and neurological disorders should identify SCC and suspect, beside trauma or infectious disease, an epidural mass. An urgent oncologist pediatric evaluation should be requested following SCC discovery. Prompt diagnosis is crucial and combined with the above treatment may prevent tumor mortality, SCC morbidity and avoid unintended consequences of EDL.

## Data Availability

The data and materials of this case report are available from the corresponding author upon reasonable request.

## Abbreviations

cGy: Centigray
CT: Computed tomography
EDL: emergency decompressive laminectomy
FDG-PET: ^18^F-fluordeoxyglucose positron emission tomography
HL: Hodgkin lymphoma
L: Lumbar
MRI: Magnetic resonance imaging
NHL: Non-Hodgkin lymphoma
SCC: Spinal cord compression
Th: Thoracic

## References

[1] Gunes D, Uysal KM, Cetinkaya H, Tekin HG, Yuceer N, Sarialioglu F, Olgun N (2009). Paravertebral malignant tumors of childhood: analysis of 28 pediatric patients. Childs Nerv Syst.

[2] Klein SL, Sanford RA, Muhlbauer MS (1991). Pediatric spinal epidural metastases. J Neurosurg.

[3] Huisman TAGM (2009). Pediatric tumors of the spine. Cancer Imaging.

[4] Gupta V, Srivastava A, Bhatia B (2009). Hodgkin disease with spinal cord compression. J Pediatr Hematol Oncol.

[5] Tantawy AAG, Ebeid FSE, Mahmoud MA, Shepl OE (2013). Spinal cord compression in childhood pediatric malignancies: multicenter egyptian study. J Pediatr Hematol Oncol.

[6] Toto RL, Zuckerbraun NS, Manole MD (2016). Neck pain in a 12-year-old female: an unusual diagnosis. J Emerg Med.

[7] Grimm S, Chamberlain M (2011). Hodgkin's lymphoma: a review of neurologic complications. Adv Hematol.

[8] Higgins SA, Peschel RE (1995). Hodgkin's disease with spinal cord compression. A case report and a review of the literature. Cancer.

[9] Aysun S, Topçu M, Günay M, Topaloǧlu H (1994). Neurologic features as initial presentations of childhood malignancies. Pediatr Neurol.

[10] Pollono D, Tomarchia S, Drut R, Ibañez O, Ferreyra M, Cédola J (2003). Spinal cord compression: a review of 70 pediatric patients. Pediatr Hematol Oncol.

[11] Baroni L, Fornaciari S, Predieri B, Bergonzini P, Guerra A, Paolucci P, Iughetti L (2014). Paraplegia by spinal cord compression as the initial manifestation of Hodgkin's disease: a case report. Acta Biomed.

[12] Ghedira K, Matar N, Bouali S, Zehani A, Boubaker A, Jemel H (2019). Hodgkin lymphoma revealed by epidural spinal cord compression. J Spinal Cord Med.

[13] Abu-Bonsrah N, Boah AO, Goodwin CR, Larman T, Crane GM, Sciubba DM (2016). Epidural spinal compression as an initial presentation of Hodgkin lymphoma. J Clin Neurosci.

[14] Xu Y, Wang J, Peng Y, Zeng J (2010). CT characteristics of primary retroperitoneal neoplasms in children. Eur J Radiol.

[15] Kluge R, Kurch L, Georgi T, Metzger M (2017). Current role of FDG-PET in pediatric Hodgkin’s lymphoma. Semin Nucl Med.

[16] Cheah CY, Bröckelmann PJ, Chihara D, Moskowitz AJ, Engert A, Jerkeman M, el-Galaly TC, Augustson B, Vose J, Bartlett NL, Villa D, Connors JM, Feldman T, Pinnix CC, Milgrom SA, Dabaja B, Oki Y, Fanale MA (2016). Clinical characteristics and outcomes of patients with Hodgkin lymphoma with central nervous system involvement: an international multicenter collaboration. Am J Hematol.

[17] McGirt MJ, Chaichana KL, Atiba A (2008). Incidence of spinal deformity after resection of intramedullary spinal cord tumors in children who underwent laminectomy compared with laminoplasty. J Neurosurg Pediatr.

[18] Coccia PF, Pappo AS, Beaupin L (2018). Adolescent and young adult oncology, version 2.2018, NCCN clinical practice guidelines in oncology. J Natl Compr Cancer Netw.

[19] Hughes N, Stark D (2018). The management of adolescents and young adults with cancer. Cancer Treat Rev.

